# Extracellular degradation of lipoprotein lipase in rat adipose tissue

**DOI:** 10.1186/1471-2121-6-4

**Published:** 2005-01-25

**Authors:** Gengshu Wu, Gunilla Olivecrona, Thomas Olivecrona

**Affiliations:** 1 Department of Medical Biosciences, Physiological Chemistry, Umeå University, SE-90187 Umeå, Sweden; 2 Signal Transduction Research Group, Department of Biochemistry, University of Alberta, Edmonton, Alberta, T6G 2S2, Canada; 3 Umeå University, Physiological Chemistry, SE-90187 Umeå, Sweden

## Abstract

**Background:**

Recent studies *in vivo *indicate that short-term regulation of lipoprotein lipase (LPL) in rat adipose tissue is post-translational and occurs by a shift of the lipase protein towards an inactive form under the influence of another gene with short-lived message and product. It has not been possible to reproduce this process with isolated adipocytes suggesting that other cells are needed, and perhaps mediate the regulation. The objective of the present study was, therefore, to explore if explants of adipose tissue could be used for studies of the regulatory process.

**Results:**

When explants of rat epididymal adipose tissue were incubated, LPL mass and activity decreased rapidly. Mass and activity within adipocytes remained constant for at least six hours, demonstrating that it was the extracellular portion of the enzyme that decreased. Adipocytes isolated from the explants after three or six hours of incubation retained their ability to secrete LPL to the medium. Addition of a cocktail of protease inhibitors to the incubation medium slowed down the decrease of LPL mass. Chloroquine was without effect, indicating that the degradation was not lysosomal. ^125^I-labeled LPL added to the medium was degraded to acid soluble products, indicating that the degradation occurred extracellularly. Fragmentation of the labelled lipase occurred in conditioned medium and this process was virtually abolished by two MMP inhibitors.

**Conclusions:**

The decrease of LPL mass and activity that occurs when explants of rat adipose tissue are incubated is due to proteolysis of extracellular LPL. The adipocytes continue to produce and secrete the enzyme. The effect of inhibitors indicates, but does not prove, that the degradation is mediated by MMPs. It appears that this process is accelerated in the tissue fragments compared to intact tissue.

## Background

Lipoprotein lipase (LPL) hydrolyzes triglycerides in very low-density lipoproteins and chylomicrons [1]. Tissue-specific regulation of LPL activity is a major mechanism to distribute lipids among tissues according to the physiological needs [2]. Current information indicates that in adipose tissue, the regulation is post-translational and occurs by a shift of the lipase protein towards an inactive form under the influence of another gene with short-lived message and product [3]. This information derives from *in vivo *experiments. To study the underlying mechanism an *in vitro *model is urgently needed. Experiments with isolated adipocytes do not seem to bring out the mechanism and the *in vivo *experiments indicate that it is the extracellular LPL that is the target for the regulation [4]. We have therefore explored the possibility to use tissue explants and report our experiences in this paper. The results support the view that it is the extracellular enzyme that is being regulated. Unfortunately the preparation of tissue explants seems to trigger a proteolytic response in the tissue.

## Results

### LPL activity and mass decreased when explants of rat adipose tissue were incubated

**Figure 1:**
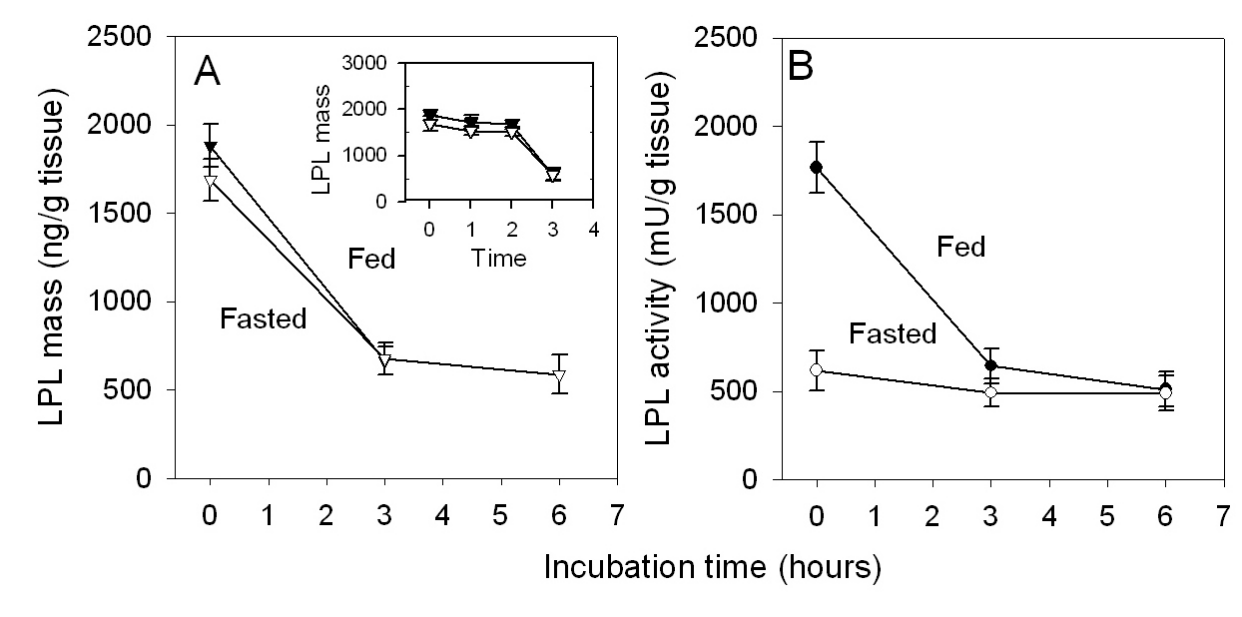
**Changes in LPL mass and activity during incubation of adipose tissue explants. **Epididymal adipose tissue was dissected from fed and 24 h-fasted rats, cut into small pieces and rinsed in cold PBS. About 100 mg tissue was then incubated at 37°C for the indicated times as described in the Methods section. LPL mass (triangles, panel A) and activity (circles, panel B) in tissue explants from fed (filled symbols, ● and ▼) or fasted (open symbols, ○ and ▽) rats. Values at the start of the incubations were for LPL mass: 17.4 ± 1.1 and 17.1 ± 2.5 ng/μg DNA and for LPL activity: 6.3 ± 1.8 and 3.5 ± 0.7 mU/μg DNA, in tissues from fed and fasted rats, respectively. The inset shows a separate experiment where incubations were stopped at shorter times. Only LPL mass was followed in that experiment. Some of the data points for fed and fasted rats fall on top of each other. Values are mean ± SEM for five parallel incubations.

In the first set of experiments we incubated explants of rat adipose tissue and followed LPL mass and activity (Figure 1). LPL mass decreased by more than 50% in three hours. The decrease then continued so that after six hours only around 20% of the original mass remained. With tissues from fed rats, in which most of the LPL protein is in the catalytically active form, the LPL activity decreased in parallel to LPL mass. In tissues from fasted rats, in which most of the LPL protein is in the catalytically inactive form, the decrease was less steep for LPL activity than for LPL mass. The inset in Figure 1 shows that there was a delay of about two hours before the decrease of LPL mass accelerated. There was no difference between tissue from fed and fasted rats.

We tried variations in technique and several different incubation media in experiments such as those in Figure 1. There was some variation in the absolute values but the results were in principle the same, a rapid decline of LPL mass and activity. The rate at which LPL mass decreased was similar with tissue explants from fed and fasted rats, and LPL activity roughly followed LPL mass in tissues from fed rats.

One possible explanation could be that LPL was released from the tissue into the medium. The amounts of LPL mass or activity that appeared in the medium were, however, small (Table 1). To test whether lipase might have adsorbed to the plastic dishes these were rinsed out with warm SDS solution, but only small amounts of LPL protein were recovered (Table 1). Hence it is clear that there was a loss of LPL mass from the system.

**Table 1:** Changes in LPL mass during incubation. Explants of rat adipose tissue were incubated for three hours as in Figure 1. The tissue explants and the medium were recovered and then the vessel was rinsed out with warm (~80°C) SDS solution. This was then suitably diluted with Triton X-100 to match the composition of the medium used for the ELISA. Mean ± SEM of five parallel incubations.

	LPL mass (ng/g tissue)	
	Before incubation	After incubation

Adipose tissue	1882 ± 13	665 ± 11*
Culture medium		26 ± 6
Washing solution		12 ± 3
Total	1882 ± 13	702 ± 8 *

* P < 0.001 compared with before incubation

**Figure 2:**
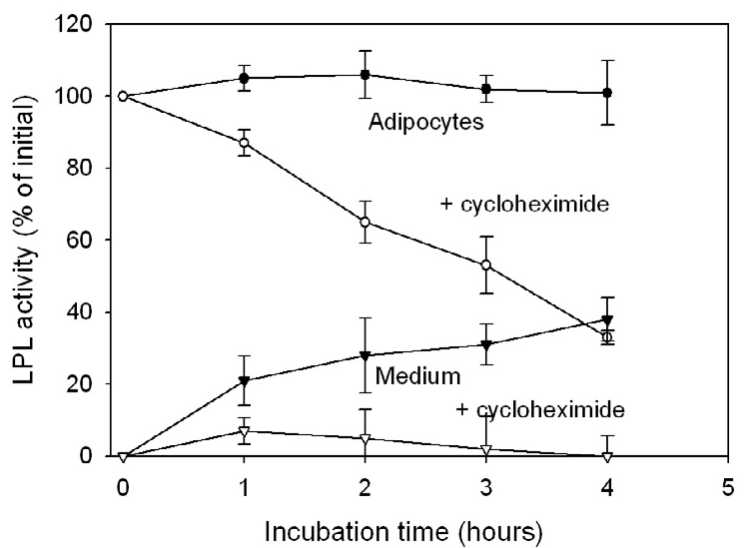
**LPL activity in cells and in medium during incubation of isolated adipocytes, and the effect of heparin. **Adipocytes (from 180 – 200 g rats) were incubated under the same conditions as used for the tissue explants in Figure 1 without (filled symbols) or with (open symbols) 0.1 mg/ml cycloheximide. ●, ○ – adipocytes; ▼, ▽ – medium. Mean ± SEM for five wells at each time.

Another possibility was that the cells lost their ability to produce LPL. Isolated adipocytes, incubated under the same conditions as the tissue explants, released LPL activity (Figure 2) and mass (data not shown) to the medium, while cellular activity (Figure 2) and mass (data not shown) increased slightly. Total LPL activity in the system increased by about 60% during four hours of incubation. When cycloheximide was added, the release of LPL to the medium was virtually abolished and cellular LPL activity decreased with time. Total LPL activity in the system decreased by almost 70%. This demonstrates that the cells depend on synthesis of new LPL protein to sustain LPL activity and secretion to the medium. Adipocytes isolated from tissue explants that had been incubated for three or six hours as in Figure 1 retained the ability to release LPL to the medium (not shown). Hence, the loss of LPL activity that occurred when tissue explants were incubated was not due to a loss of LPL production within adipocytes. In these experiments we also noted that the release of LPL from adipocytes was similar whether the cells were isolated from fed or fasted rats (not shown).

### Is the loss of LPL mass an intra- or extracellular event?

**Figure 3:**
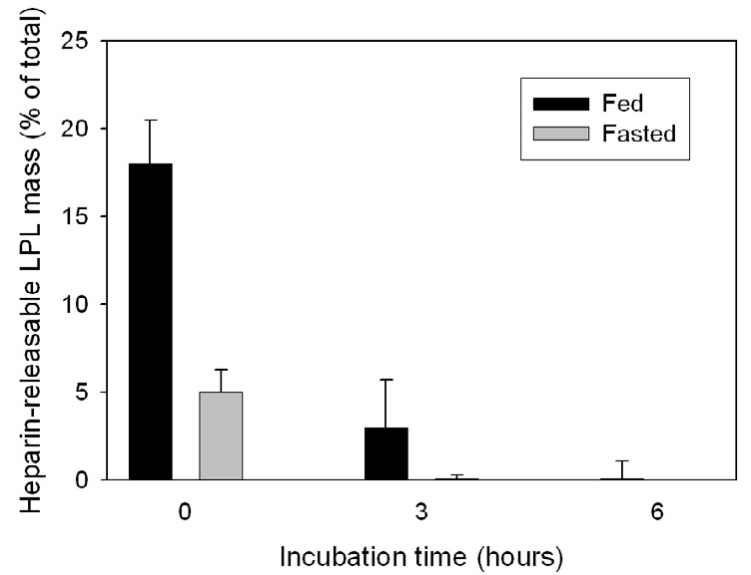
**Changes in the amount of heparin-releasable LPL mass during the incubation. **Conditions as in Figure 1 but at the designated times, the explants of adipose tissue were transferred to new medium containing heparin and incubated for a further 45 min. Values are means ± SEM for five parallel incubations. Black bars represent explants from fed rats; grey bars represent explants from fasted rats.

**Figure 4:**
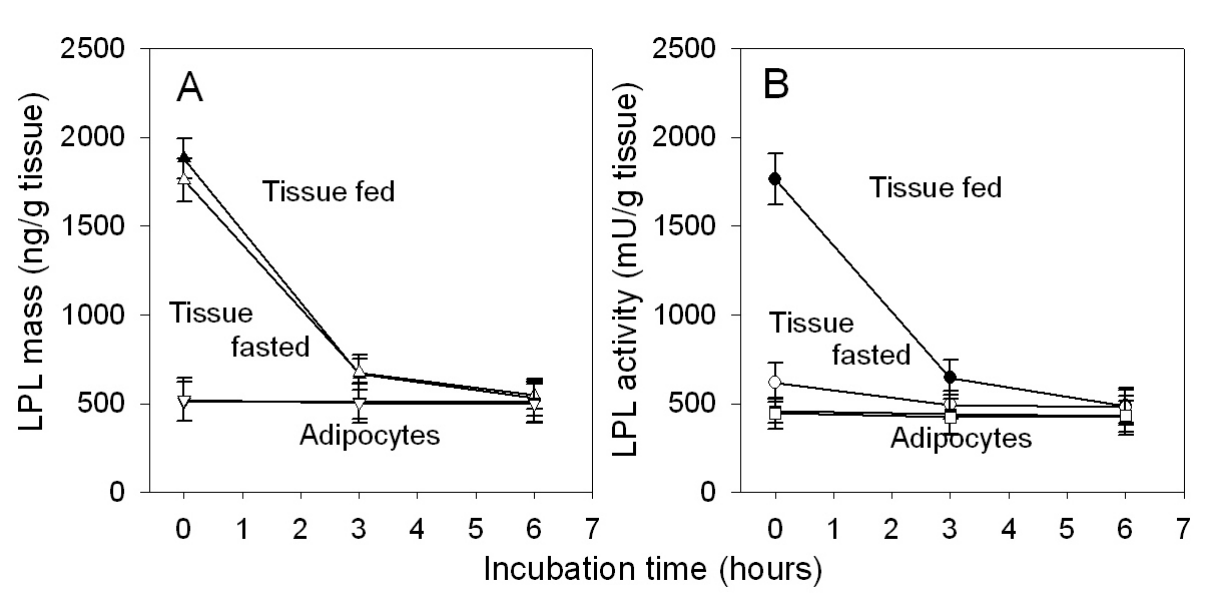
**LPL activity and mass within and outside the adipocytes. **Conditions as in Figure 1 but adipose tissue explants from 180–200 g rats were used to get enough material to isolate adipocytes. At the end of the incubation some of the tissue explants were incubated with collagenase and adipocytes were isolated as described in the methods section. Filled symbols – fed rats; open symbols – fasted rats. Panel A shows LPL mass. ▲, △ – tissue, ▼, ▽ – adipocytes. Panel B shows LPL activity. ■, □ – tissue, ●, ○ – adipocytes. Values are means ± SEM for five parallel incubations. Some of the data points for fed and fasted rats fall on top of each other.

Heparin release is often used to assess the LPL activity of tissues and releases mainly extracellular LPL [4]. Figure 3 shows that in fresh explants of adipose tissue a substantial fraction of tissue LPL could be released by heparin. More LPL was released from tissues of fed rats (Figure 3). When the tissue explants were incubated for three hours before the heparin challenge, much less LPL was released and after six hours virtually no LPL was released (Figure 3). This suggested that the decrease of LPL affected mainly the extracellular enzyme. To test this hypothesis we isolated adipocytes from tissue explants after three or six hours of incubation (Figure 4). The LPL activity and mass in the adipocytes was the same when the cells were isolated from tissue explants that had been incubated for three or six hours as when they were isolated from fresh tissue explants. Hence, it was extracellular LPL (calculated as the difference between tissue total and adipocytes) that accounted for the rapid decrease of tissue LPL during incubation.

### Is the LPL protein degraded?

**Figure 5:**
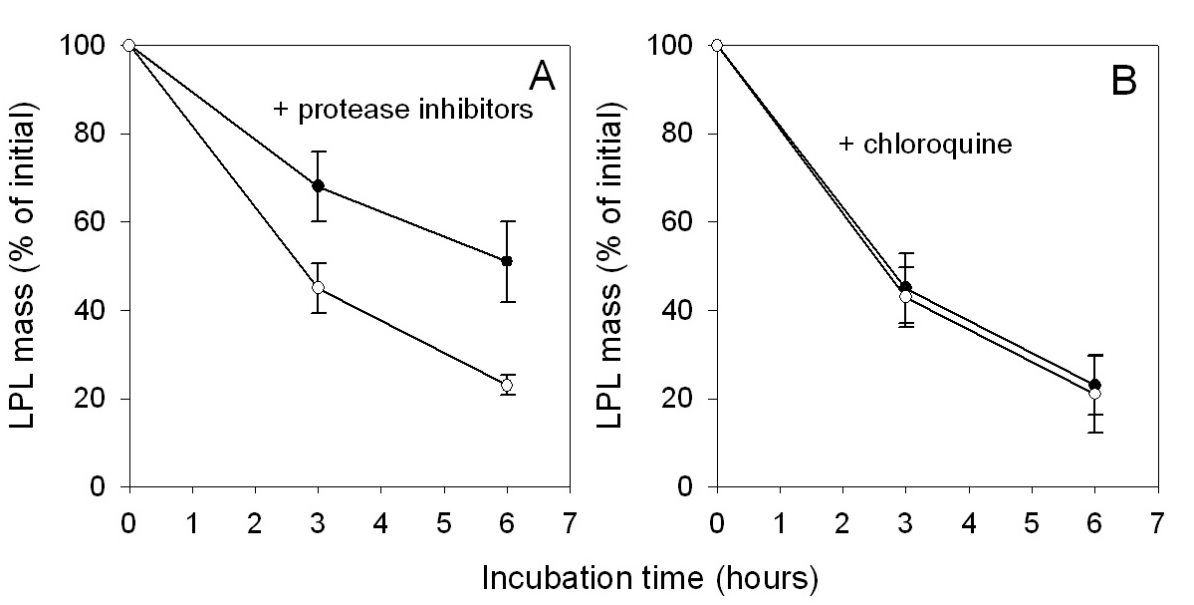
**Effect of protease inhibitors on the decrease of LPL mass during incubation of tissue explants. **Conditions as in Figure 1. A cocktail of protease inhibitors (panel A) or chloroquine (final concentration 150 mM, panel B) were included in the medium of some of the incubations. The tissue was from fed rats. Similar results (not shown) were obtained with tissue from fasted rats. ○ – without protease inhibitor, ● – with protease inhibitor. Mean ± SEM for five parallel incubations.

These results indicated that the decline of LPL mass occurred through proteolytic cleavage of the extracellular enzyme. To test this hypothesis, we included a cocktail of protease inhibitors in the medium used for incubation of tissue explants. This slowed down the decrease of LPL mass (Figure 5A). Chloroquine had no effect (Figure 5B), indicating that the degradation did not occur in lysosomes.

**Figure 6:**
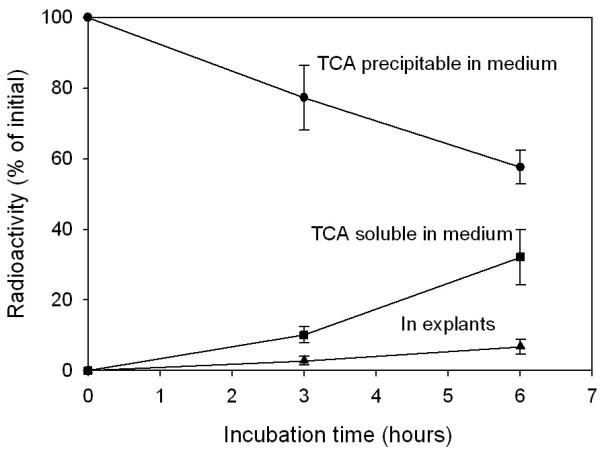
**Fate of ^125^I-LPL added to the incubation. **Conditions as in Figure 1 but ^125^I-LPL was added to the medium. ● – TCA precipitable radioactivity in medium, ▲ – TCA precipitable radioactivity in the tissue explants, ■ – TCA soluble radioactivity in medium. Mean ± SEM for five parallel incubations.

**Figure 7:**
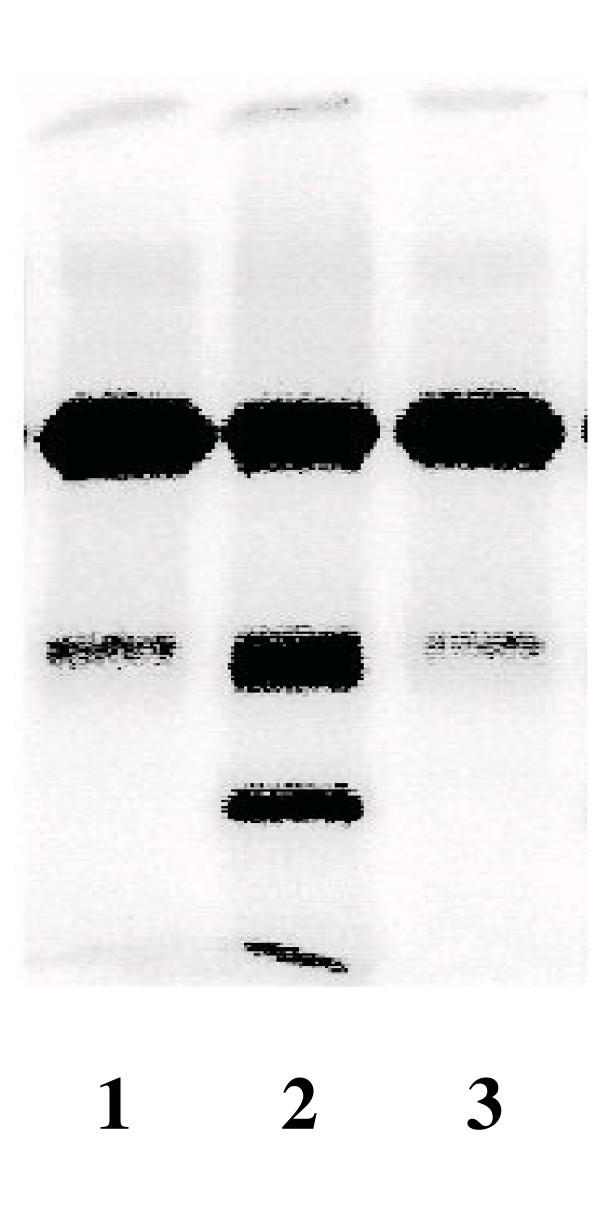
**Analysis by SDS-PAGE of the cleavage of ^125^I-LPL in conditioned medium and the effect of an MMP inhibitor **Explants of adipose tissue from fed rats were incubated as in Figure 1 for four hours and the medium was collected. ^125^I-LPL was then incubated at 4°C (to minimize the risk of conformational changes) in this medium with or without the MMP inhibitor GM6001 (5 μg/ml). Lane 1 – fresh medium, lane 2 – conditioned medium, lane 3 – conditioned medium + inhibitor. The lower band in lane 1 is a proteolytic fragment of LPL that is always present in preparations of the enzyme from bovine milk [23].

To further study the proteolytic process, ^125^I-LPL was added to the incubations. TCA soluble material appeared in the medium demonstrating that proteolytic degradation took place (Figure 6). We noted that some of the TCA precipitable material became associated with the tissue explants suggesting, binding and/or uptake of the lipase. To explore if the degradation required that the lipase was taken up into cells in the tissue we incubated the labelled lipase in conditioned medium. Analysis by SDS-PAGE showed that several fragments were formed (Figure 7). Addition of either of two non-specific MMP inhibitors, Captopril or GM6001, prevented the degradation almost completely.

## Discussion

The objective for this study was to find an *in vitro *system to study the mechanism for down-regulation of LPL activity in rat adipose tissue that occurs on food deprivation. Isolated adipocytes have been tried in several laboratories [5-9], but the differences with nutritional state are rather small. This is true whether one measures LPL within the cells or the rates at which the cells secrete LPL to the medium. These observations are repeated here. The lack of difference within adipocytes indicates that other cell types are needed and may in fact be responsible for the pronounced down-regulation of extracellular LPL activity that occurs on food deprivation [3,4]. We therefore tried to use tissue explants, which have proved valuable in other studies of adipose tissue [10]. Our results show that degradation of the enzyme was a major process when explants were incubated. The degradation occurred extracellularly; LPL mass and activity in the adipocytes did not change during incubation for up to six hours. Added ^125^I-LPL was degraded and this was prevented by addition of MMP inhibitors to the medium.

There must be damage of cells when the tissue is cut into small pieces, and there is probably some degree of hypoxia during incubation of the pieces. Cultured explants have, however, been widely and successfully used to explore various aspects of adipose tissue biology ([10] and references therein). In preliminary experiments we found that the explants retained their ability to take up glucose, to synthesize proteins and to secrete leptin. Adipocytes isolated from the explants after several hours of incubation had the same LPL activity as adipocytes from fresh tissue (Figure 4). This indicates that the cells produced LPL at an essentially unchanged rate, since the LPL activity in adipocytes decreased rapidly when protein synthesis was inhibited by cycloheximide (Figure 2). Hence, the rapid decrease of LPL when explants were incubated was not due to a general loss of functionality, but reflected specific processes leading to degradation of extracellular LPL.

Our results are in line with observations made already in the sixties. There was evidence from chromatographic separations for at least two different forms of LPL in rat adipose tissue [11]. Cunnigham and Robinson found that incubation of fat pads from fed rats resulted in a rapid loss of LPL activity until a low activity, stable to prolonged incubation, was attained [12]. In contrast, the LPL activity of isolated fat cells was stable to prolonged incubation. The concept of stable and unstable forms of the lipase can now be interpreted as a reflection of extracellular lipase that is exposed to proteolysis, and intracellular lipase that is protected from the extracellular proteases.

The degradation of LPL in conditioned medium was almost completely abolished by the two non-specific MMP inhibitors that we tested. We have not characterized the proteolytic activity further but note that adipose tissue produces at least two MMPs, 2 and 9 [13]. The loss of LPL mass during incubation of tissue explants was relatively slow during the first two hours and then accelerated. It is likely that the tissue trauma and/or the loss of blood circulation triggered an activation of the MMP system. It has been shown that primary culture of human adipose tissue explants dramatically alters adipocyte gene expression [14]. It is of interest to note that LPL activity does not decrease during perfusion of fat pads [15], whereas it does decrease when whole fat pads are cut out and incubated *in vitro *[12].

Two pathways for turnover of adipose tissue LPL have been demonstrated so far. One is dissociation of the lipase, perhaps after loss of catalytic activity, into the blood and degradation in the liver [16]. Release of LPL into blood from adipose tissue has been directly demonstrated by measurement of arterio-venous difference in man [17]. This pathway can, however, not operate in tissue explants. Another pathway, demonstrated with cultured fat cells, is endocytosis and degradation in lysosomes [18]. This pathway did not seem to contribute significantly in the present system since the rate at which LPL mass decreased was not affected by chloroquine. The present findings suggest a third pathway, extracellular proteolysis in the tissue.

## Conclusions

The rapid decrease of LPL that occurs when adipose tissue explants are incubated engages only the extracellular enzyme. The adipocytes continue to produce and secrete the enzyme and intracellular LPL remains essentially constant for at least six hours.

The decrease in extracellular LPL is due to proteolytic cleavage/degradation of both active and inactive forms of the enzyme.

The effects of inhibitors indicate, but do not prove, that the degradation is mediated by MMPs. It appears that this process is accelerated in the tissue fragments compared to intact tissue.

## Methods

### Animals

Male Sprague-Dawley rats were from Möllegaard Breeding Center (Ejby, Denmark). Unless otherwise stated, the rats were 23 days old and weighed around 60 g. After transport to Umeå they were allowed to acclimatize for seven to ten days by which time they had reached a weight of approximately 120 g. The rats were kept in a well ventilated, temperature (21°C) and humidity (40–45%) controlled room with free access to a standard laboratory chow (Laktamin AB, Stockholm) and tap water. The light in the room was on between 6 a.m. and 6 p.m. In experiments where the rats were to be fasted, food was withdrawn from the cages at 6 a.m. and a grid was placed at the bottom of the cages to prevent coprophagia. The adipose depot used in all experiments was the periepididymal one. The rats were killed by decapitation. Animal experiments were approved by the animal ethics committee in Umeå.

### Materials

Cycloheximide, bovine serum albumin (BSA), the MMP inhibitors GM6001 (Galardin) and Captopril, chloroquine and collagenase were from SIGMA (St. Louis, MO, USA). Protease inhibitor cocktail tablets "Complete Mini" were from Roche Diagnostics, Mannheim, Germany. Heparin was from Lövens (Malmö, Sweden). Substrate for the LPL activity assay was ^3^H-labelled triolein in Intralipid (10%) kindly prepared by Pharmacia-UpJohn (Stockholm, Sweden). Parker medium (Parker 199) was from SBL (Stockholm, Sweden). ^125 ^I-LPL was prepared as before [19]. All other reagents were of the highest commercial grade possible.

### Assays

LPL was extracted from tissues by homogenization in a Tris-HCL buffer (pH 8.2) containing detergents and protease inhibitors as described [20]. The homogenate was centrifuged for 15 min at 3000 rpm after which the intermediate phase (between the floating fat droplets and the pellet) was used for assay of LPL activity and mass. In most cases the extract was kept on ice and assayed within a few hours. Under these conditions LPL activity is stable. In some cases the extracts were frozen and kept at -70°C for later assay.

LPL activity was measured as described previously [20]. Briefly, two μl of tissue homogenate (triplicate samples) was incubated for 60 min at 25°C with substrate in the presence of ten μl heat-inactivated serum from fasted rats (as source of apolipoprotein CII) and 6% BSA. The total volume was 200 μl. After termination of lipolysis by addition of organic solvents, the fatty acids were extracted and counted for radioactivity. One mU of lipase activity represents one nmol of fatty acids released per minute.

LPL mass was measured with an ELISA as described [20]. The chicken antibodies used recognize both active and inactive forms of the lipase [21]. Briefly, three different dilutions of tissue homogenate were incubated in microtiter plate wells previously coated with affinity-purified chicken anti-LPL IgG. Detection was mediated via the 5D2 monoclonal antibody (a kind gift by Dr John Brunzell, University of Washington, Seattle) followed by a peroxidase conjugated anti-mouse IgG antibody. Absorbance at 490 nm was measured in a Spectramax microplate spectrophotometer (Molecular Devices, Sunnyvale, CA, USA).

DNA content was assayed using Labarca's method [22].

### *In vitro *incubation of adipose tissue

Epididymal adipose tissue was dissected out from fed or 24 h fasted rats. The tissue was cut into small pieces (5 mg or less). A total of about maximal 100 mg tissue pieces were immediately put into culture plates. Each well contained 15 ml of Parker Medium 199 supplemented with 2% BSA, 0.5% casein hydrolysate, 10 mM glucose and adjusted to pH 7.4. Incubations were at 37°C and 5 % CO_2_: 95 % O_2 _with continuous gentle shaking motion in a Cellstar Incubator (Queue Systems, Asheville, Canada). After incubation, the tissues were prepared for measurement of LPL activity and mass as described above. In some experiments samples of the medium were taken for assay of LPL activity and/or mass.

In some experiments adipocytes were prepared after collagenase treatment of the tissue pieces as described [4]. To measure heparin releasable LPL (HR-LPL), adipose tissue explants were incubated with heparin (final concentration was 50 IU/ml) for 45 min at 37°C.

### Statistics

Student's t-test was used for analysis of the data.

## Authors' contributions

GW carried out the experiments and participated in their design and in writing of the manuscript, TO participated in the design of the study and drafted the manuscript. GO conceived of the study and coordinated the work. All authors read and approved the final manuscript.
